# Biosynthesis of silver nanoparticles using *Haloferax* sp. NRS1: image analysis, characterization, in vitro thrombolysis and cytotoxicity

**DOI:** 10.1186/s13568-021-01235-3

**Published:** 2021-05-26

**Authors:** Hend M. Tag, Amna A. Saddiq, Monagi Alkinani, Nashwa Hagagy

**Affiliations:** 1 Department of Biology, College of Science and Arts At Khulis, University of Jeddah, Jeddah, Saudi Arabia; 2 Department of Biology, College of Science, University of Jeddah, Jeddah, Saudi Arabia; 3 Department of Computer Science, College of Computing and Information Technology, University of Jeddah, Jeddah, Saudi Arabia; 4 Botany & Microbiology Department, Faculty of Science, Suez Canal University, Ismailia, 41522 Egypt

**Keywords:** Biosynthesis, Silver-nanoparticles, *Haloferax* sp, Thrombolysis, Cytotoxicity

## Abstract

*Haloferax* sp strain NRS1 (MT967913) was isolated from a solar saltern on the southern coast of the Red Sea, Jeddah, Saudi Arabia. The present study was designed for estimate the potential capacity of the *Haloferax* sp strain NRS1 to synthesize (silver nanoparticles) AgNPs. Biological activities such as thrombolysis and cytotoxicity of biosynthesized AgNPs were evaluated. The characterization of silver nanoparticles biosynthesized by *Haloferax* sp (*Hfx*-AgNPs) was analyzed using UV–vis spectroscopy, transmission electron microscopy (TEM), X-ray diffraction (XRD), and Fourier-transform infrared spectroscopy (FTIR). The dark brown color of the *Hfx*-AgNPs colloidal showed maximum absorbance at 458 nm. TEM image analysis revealed that the shape of the *Hfx*-AgNPs was spherical and a size range was 5.77- 73.14 nm. The XRD spectra showed a crystallographic plane of silver nanoparticles, with a crystalline size of 29.28 nm. The prominent FTIR peaks obtained at 3281, 1644 and 1250 cm^− 1^ identified the Functional groups involved in the reduction of silver ion reduction to AgNPs. Zeta potential results revealed a negative surface charge and stability of *Hfx*-AgNPs. Colloidal solution of *Hfx-*AgNPs with concentrations ranging from 3.125 to 100 μg/mL was used to determine its hemolytic activity. Less than 12.5 μg/mL of tested agent showed no hemolysis with high significant decrease compared with positive control, which confirms that *Hfx*-AgNPs are considered non-hemolytic (non-toxic) agents according to the ISO/TR 7405-1984(f) protocol. Thrombolysis activity of *Hfx*-AgNPs was observed in a concentration-dependent manner. Further, *Hfx*-AgNPs may be considered a promising lead compound for the pharmacological industry.

## Key points

- The haloarchaeon Haloferax sp. strain NRS1 (MT967913), isolated from solar saltern in Jeddah, Saudi Arabia, has promising ability when synthesized with silver nanoparticles.
- Characterization of silver nanoparticles (AgNPs) synthesized by haloarchaeon *Haloferax* sp. strain NRS1 was performed by UV–vis spectroscopy, transmission electron microscope (TEM), X-ray diffraction (XRD), Fourier-transform infrared spectroscopy (FTIR).
- The low hemolytic activity exhibited by *Hfx*-AgNPs, confirmed their promising role as nano-drug delivery agents. *Hfx*-AgNPs displayed clot lysis’s properties which may be attributed to the activation of the cascade reaction of clot lysis.

## Introduction

*Haloarchaea* may be the best candidates for synthesizing nanoparticles because to S-layer glycoproteins present in their membrane. The unique property of haloarchaeal cells to quickly lyse at low concentrations of salt, leading to intracellular and membrane components being released, allowing S-proteins recovery efficient in cost. S-layer proteins of haloarchaea serve as building blocks for the synthesis of main biomolecules (proteins, lipids, glycans, nucleic acids, or their combinations) which are appropriate for nanobiotechnology (Sleytr et al. 2014). Hence, the intracellular biosynthesis of nanoparticles in haloarchaea, suggests these extremophilic microorganisms are promising in the field of nanobiotechnology (Beeler and Singh 2016).

Nanoparticles are promising in different fields such as biomedicine, drug delivery, bio-imaging, bio-labeling, photovoltaics, photocatalysis, solar cells, and data storage (Bera et al. 2010; Garcia 2011; Issa et al. 2013; Javed et al. 2020; Aisida et al. 2019a, b, c, 2021a, b). Biosynthesis of nanoparticles by microorganisms is a non-toxic and eco-friendly method (Srivastava et al. 2014). Biosynthesized nanoparticles by prokaryotes have attracted significant attention in the biomedical field because of their biocompatibility, nontoxicity **(**Abdullaeva 2017), and antimicrobial nature (Aisida et al. 2021c, d, e)**.** Depending on microorganisms' metabolic activity and nature, the nanoparticle's biosynthesis can either be an intra- or extracellular process. *Haloarchaea* possess different metal resistance mechanisms and biosynthesis of nanoparticles, however, most are still unknown (Srivastava and Kowshik 2013). Few haloarchaeal strains *Halococcus salifodinae* BK3, *Halococcus salifodinae* BK6_,_ have been reported for intracellular synthesis of silver nanoparticles (AgNPs) with antibacterial activity against gram-positive and gram-negative bacteria (Srivastava et al. 2013, 2014). The same action has been reported by the cells of *Haloferax alexandrinus* (Patil et al. 2014). Also, Costa et al. (2020) reported the ability of *Haloferax volcanii* to synthesize silver and gold nanoparticles biosynthesis with distinct properties for nanobiotechnological applications. *Haloferax* sp playing a potential role as an *in-vitro* antioxidant and antimicrobial agent (Zalazar et al. 2019). Recently, De Castro et al. (2020) reported that haloarchaea have a biotechnological potential that still needs to be explored for its significance in pharmaceutical applications. Further studies are therefore required to explore more biosynthesized nanoparticles from microorganisms with unique properties for future applications. In this study, we report the biosynthesis of silver nanoparticles intracellularly by unclassified species of *Haloferax*, *Haloferax* sp strain NRS1, and bioactivities for AgNPs. As well, characterization of biosynthesized AgNPs by UV- vis spectroscopy, TEM, XRD, and FTIR. The main objective of this work is finding a novel application for non-toxic biosynthesized AgNPs by *Haloferax sp* strain NRS1 as a significant fibrinolytic agent.

## Materials and methods

### Site description and sampling

**Fig. 1:**
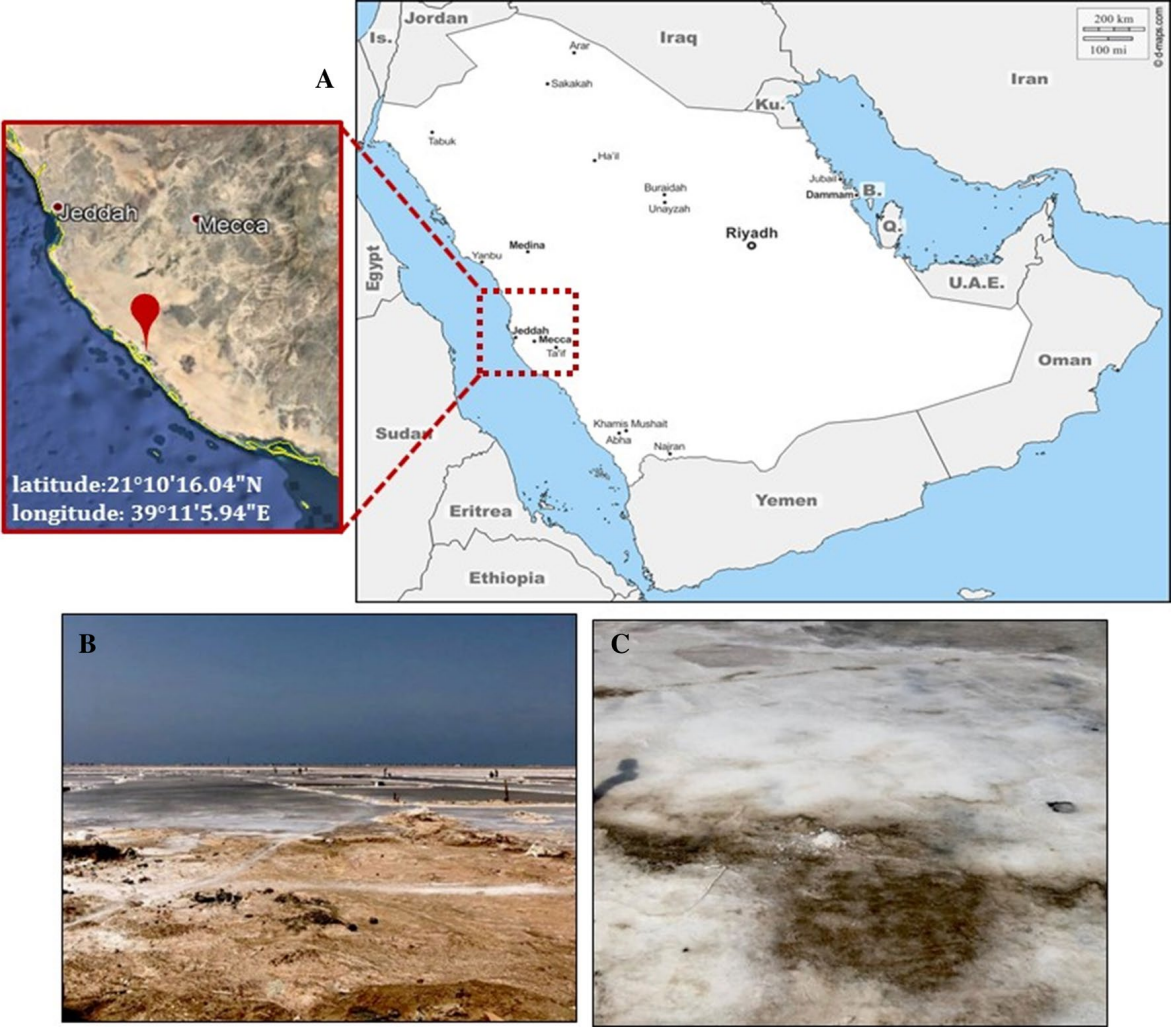
Map and **A** satellite image showing the location of the study area. **B** The studied solar saltern in the southern coast of Jeddah; C. The salt crystals were collected from the saltern

Sediment and brine samples were collected from a solar saltern located at the southern coast of Jeddah, Saudi Arabia (21^o^10′16.04''N, 39^o^11′5.94''E) in April 2019 (Fig. 1) into 1000 ml bottles of sterile Pyrex. All samples have been stored at 4 °C and upon arrival, the samples have been subjected to microbiological examination within 24 h.

### Isolation and growth conditions

Isolation of haloarchaea from sediment and brine described by Srivastava et al. (2013) which involves NTYE (25% NaCl, 0.5% tryptone, 0.3% yeast extract, 2% MgSO_4_-7H_2_O and 0.5% KCl, 2% Agar) was used. This was also in accordance to the isolation procedures described by Dyall-Smith (2008). Pure isolates were obtained by successive cultivation on NTYE and was also maintained on the same medium.

### Screening for silver-resistant haloarchaeal isolates

Five different haloarchaeal isolates, obtained after two weeks of incubation at 40 °C, were tested for the growth in the presence of AgNO_3_ in a concentration range of 0.05–1 mM according to the method described by Srivastava et al. (2014). 1 ml aliquot of cell culture was measured at 600 nm on a UV–Visible spectrophotometer (UV-2600 Series, SHIMADZU) at intervals of 24 h, culture medium without AgNO_3,_ and non-inoculated medium with AgNO_3_ considered as positive and negative control respectively.

### Silver nitrate biosynthesis

The selected haloarchaeal isolate (NRS1), kept in the dark for 10 days was inoculated in NTYE-medium supplemented with 0.5 mM AgNO3, incubated at 37 °C and stirred at 110 rpm. The synthesis of nanoparticles was observed visually by the change of biomass color from orange-red to brown-black. Then the cells were harvested by centrifugation (10,000 rpm for 20 min). The obtained pellet was washed several times with distilled water, then freeze dried at 60 °C over-night then the nanoparticles were obtained as a powder.

### Identification of the isolates

Genomic DNA was extracted from the selected isolate using a modified method from Experimental Techniques in Bacterial Genetics, described by Maloy (1990). The 16S rRNA gene was amplified with a set of *Archaea*-universal primers (Invitrogen, USA), 5'-ATTCCGGTTGATCCTGCCGG-3' primers (positions 6–25 in Escherichia coli numbering) and 5'AGGAGGTGATCCAGCCGCAG-3' primers (positions 1540–1521) reported by Ventosa et al. (2004). The PCR conditions were as follows: 50 μl of reaction system, reaction cycles 30 times, 95 °C pre-denaturation 5 min, 94 °C denaturation 1 min, 60 °C annealing 1 min, 72 °C extension 1 min 30 s, 72 °C final extension 10 min, 4 °C hold. A total of 50 ng/μl of each PCR product was used to prepare the samples delivered to MacroGen Company in Korea following their specifications. The sequences were analyzed using BLAST (http://www.ncbi.nlm.nih.gov/BLAST) to get a preliminary identify of the strain. The cluster analysis was performed using the MEGA 7software package.

### Hemolytic activity

Cytotoxicity of *Hfx*-AgNPs was evaluated through hemolytic assay according to the method described by Powell et al. (2000). To prepare erythrocyte suspension, 1 ml of fresh blood was centrifuged for five minutes at 10,000 rpm. 200 μl of the erythrocytes precipitate was added to 9.8 ml of Phosphate Buffered Saline (PBS; pH 7.4). Then the mixture was centrifuged for 15 min at 4000 rpm. The supernatant was discarded and the process repeated three times. Finally, the washed erythrocytes were diluted with PBS to make the cell suspension (2%). *Hfx*-AgNPs were prepared in six different concentrations (3.125, 6.25, 12.5, 25, 50, 100 μg/ml). Each concentration of *Hfx*-AgNPs was tested in three replicates. Aliquots of 20 µl of *Hfx*-AgNPs have aseptically placed into a microcentrifuge tube; then, RBC suspension (200 µl) was added and mixed gently. The samples were incubated at 35 °C for 60 min, then centrifuged at 3000 rpm for fifteen minutes. The absorbance (Abs) of the supernatant was read at 545 nm. PBS and Deionized water were added to erythrocyte suspension represents; the negative control (no hemolysis) and the positive control (100% hemolysis), respectively. Percentage hemolysis (%) was calculated according to the following formula (Taniyama et al. 2003).

$$ {\text{Percentage of hemolytic activity}}\; = \;\frac{{{\text{Experimental sample Abs}}\; - \;{\text{Negative control Abs}}}}{{{\text{Positive Control Abs}}}}\; \times \;100 $$

### Clot lysis

Clot lysis experiments have been conducted as previously mentioned by Prasad et al. (2006). Concisely, venous blood was drawn from the healthy volunteers, then 500 μl of blood was transferred to ten pre-weighed sterile Eppendorf. The blood was incubated for 45 min at 37 °C for allowing blood to coagulate. Then the samples were centrifuged for 15 min, at 5000 rpm. Serum has been entirely removed without disrupting the clot. Every Eppendorf has been weighed again to assess the clot's weight; 200 μl of different concentrations of synthesized NPs colloidal solution (100, 50, 25, 12.5, 6.25 and 3.125 μg/ml) were added separately. As a positive control, 200 μl of streptokinase and as a negative non-thrombolytic control, 200 μl of saline solution were separately added to the clot. All the tubes were then incubated at 37 °C for 90 min and observed for clot lysis. After incubation, the supernatant was removed, and tubes were again weighed to monitor the weight difference after clot disruption. The experiment was repeated with the blood samples of the 5 volunteers.

### Characterization of synthetized silver nanoparticles

The extinction coefficient of the synthesized *Hfx*-AgNPs colloidal solution was performed to monitor the bioreduction of silver by *Haloferax* sp strain NRS1 using a UV–Visible spectrophotometer (UV-2600 Series, SHIMADZU). Transmission electron microscopy (TEM HF-3300- Hitachi High-Tech Canada, Inc.) was used to determine the shape and size of *Hfx*-AgNPs. The size distributions from the TEM images for *Hfx*-AgNPs were further analyzed using image j Freeware Version 1.53d downloaded from the NIH website (http://rsb.info.nih.gov/ij). Zeta potential of the synthesized *Hfx*-AgNPs was determined by nanoparticle analyzer (Zetasizer Ver. 7.13, Malvern Instrument Ltd, UK). The structural characterization was determined using X-ray diffractometer (XRD Malvern Panalytical, UK). Finally, the structural units were identified with the use of Fourier Transmission Infra-Red spectroscopic (FTIR) (Perkin Elmer Spectrum GX Range Spectrometer).

### Statistical analysis

Statistical analysis was performed using the software SPSS for Windows (IBM Corporation, New York, USA). One-way ANONA test was applied for the statistical analysis for the present data according to the mathematical principles described by Kutner et al. (2005) followed by Post hoc comparisons using the Duncan's test. The result was considered to be significant when P is less than 0.05.

## Results

### Biosynthesis of silver nanoparticles

**Fig. 2:**
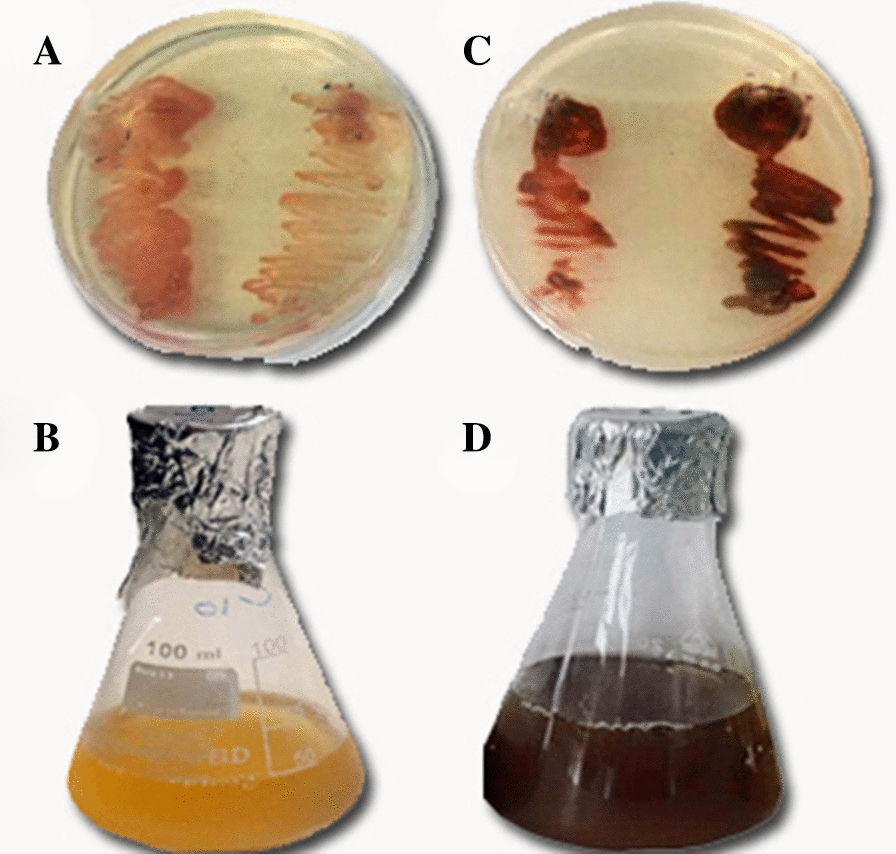
Growth of *Haloferax* sp strain NRS1 on NTYE medium in the absence and presence of 0.5 mM AgNO_3_. **A**, **B** *Haloferax* sp strain NRS1 on NTYE medium appears orange-red on agar-plate and broth respectively; **C**, **D** *Haloferax* sp strain NRS1 turned dark brown in the presence of 0.5 mM AgNO_3_ on NTYE agar and liquid media, respectively

**Fig. 3:**
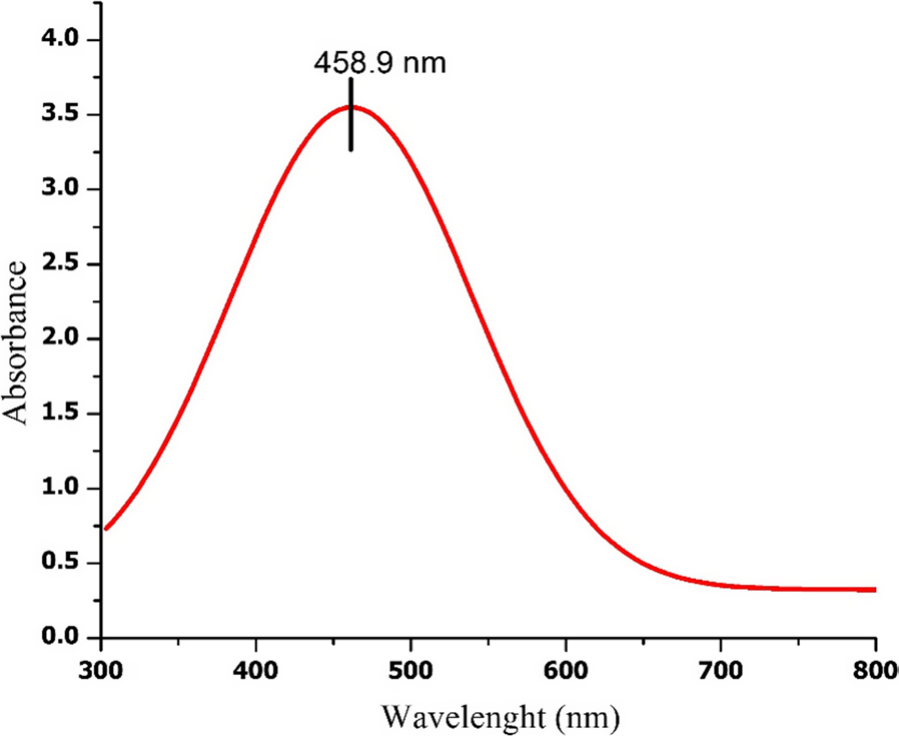
UV–Vis-Spectrum of biosynthesized AgNPs by *Haloferax sp* strain NRS1

Out of five tested isolates, one isolate (NRS1) exhibited a wide range of growth in presence of different concentrations of AgNO_3_ (0.05 up to 1 mM) and showed the highest growth rate at 0.5 mM AgNO_3_ Biosynthesis of silver nanoparticles change in the color from orange-red to brown-black was observed after one week of incubation at 37 °C, in the presence of 0.5 mM AgNO_3_. No color change was observed in control (culture medium without AgNO_3_). The appearance of brown-black color indicated the formation of AgNPs by the potential strain (Fig. 2). A peak at 458 nm in the UV–Vis spectrum further confirmed the formation of AgNPs (Fig. 3).

### Identification of the potential strain

**Fig. 4:**
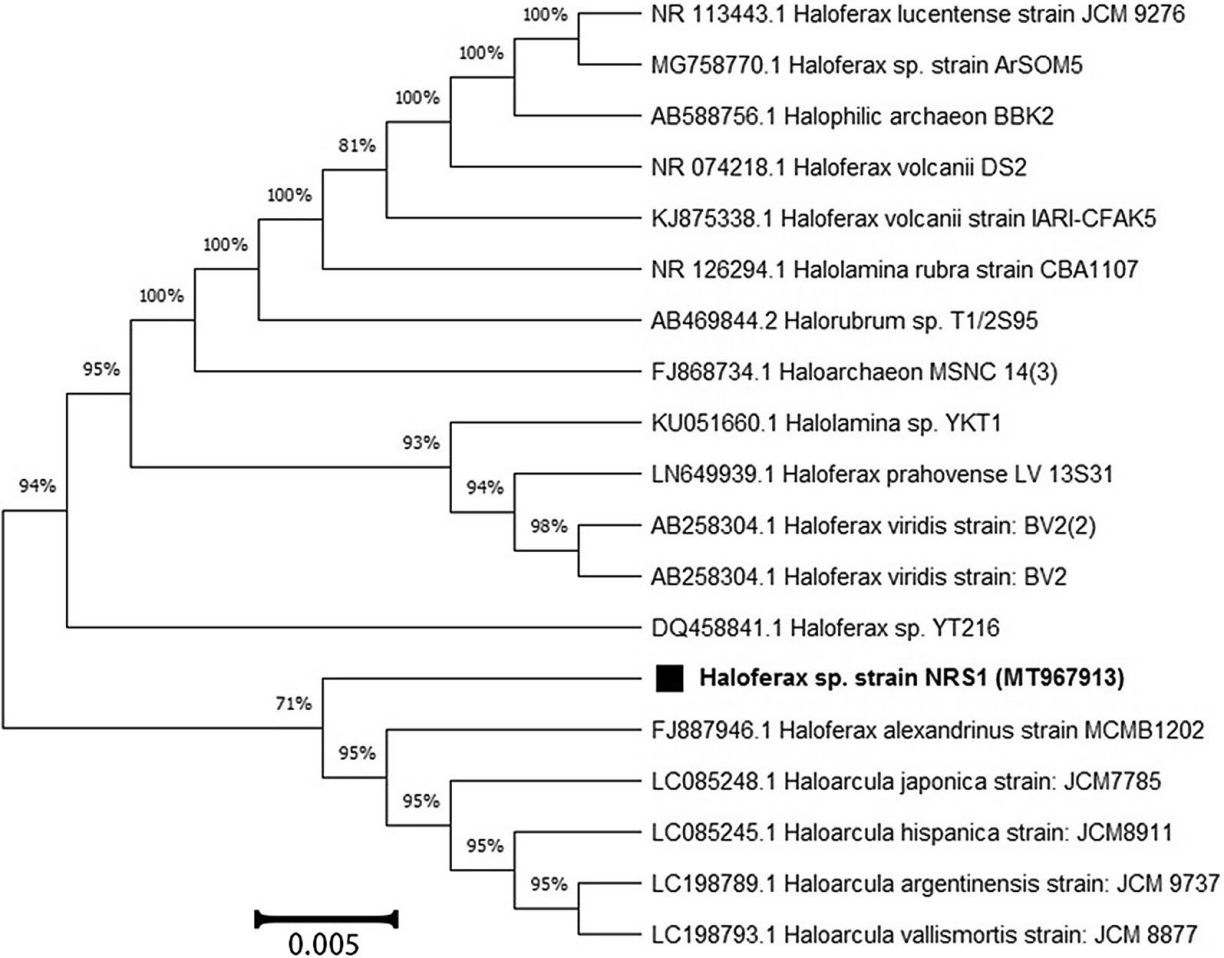
Maximum likelihood phylogenetic tree based on 16S rRNA gene sequences showing the relationship between *Haloferax sp* strain NRS1 and closely related taxa. Scale bar indicates 0.005 substitutions per nucleotide position

Analysis of 16S rRNA gene sequencing indicated that the potential strain is a member of *Haloferax* (unidentified species) with 90% sequence similarity. The 16S rRNA gene data of the haloarchaeal strain reported in this study have been deposited in the NCBI and GenBank nucleotide sequence databases under the accession number **MT967913** (Fig. 4). The strain has been deposited in the Egyptian Microbial Culture Collection (EMCC) under the code *Hfx*-NRS1 EMCC 23999.

### Characterization of silver nanoparticles

**Fig. 5:**
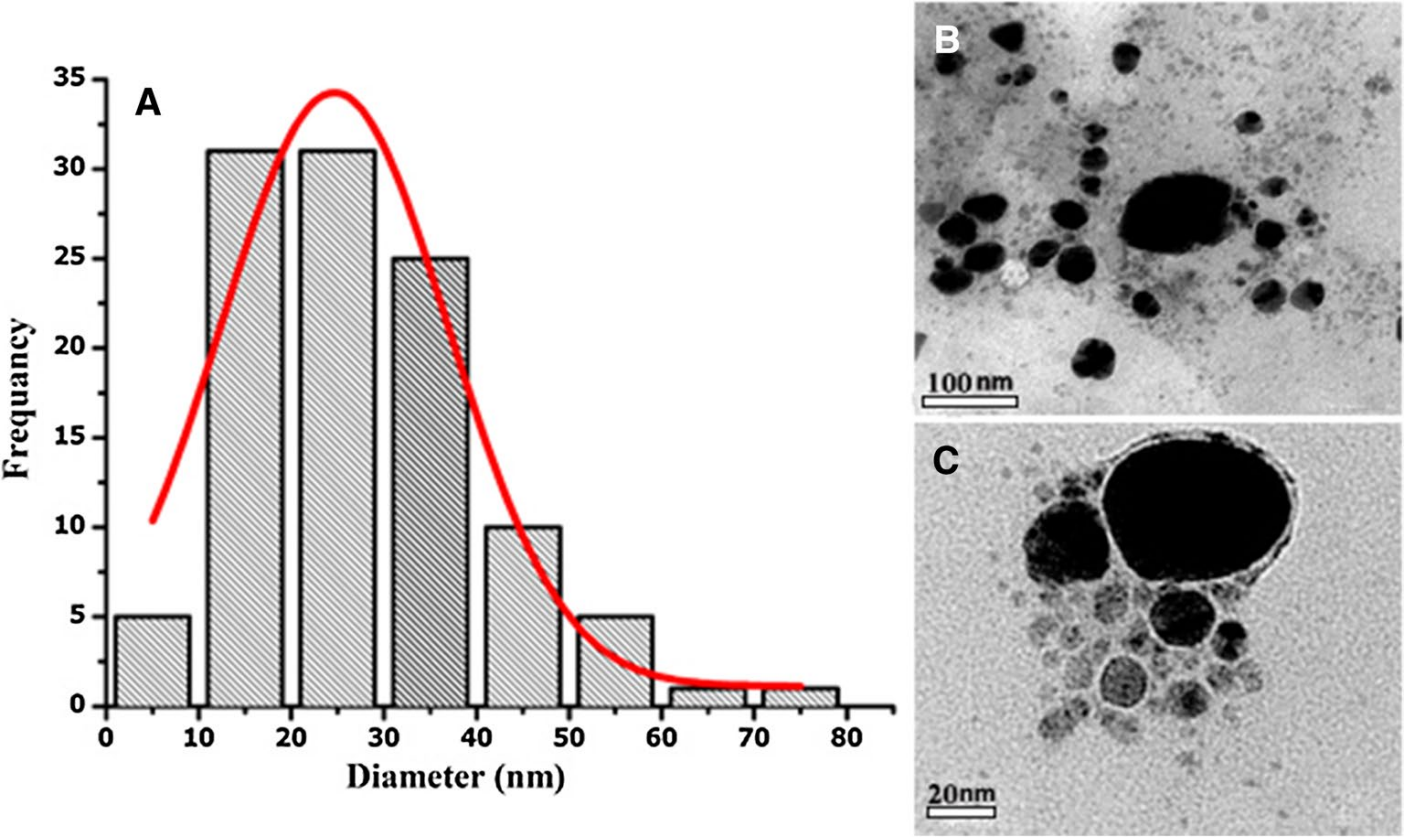
**A** Histogram of the particle diameter size distribution of the *Hfx*-AgNPs based on TEM image analysis. Red line: Gaussian distribution fit. **B** Transmission electron microscopy (TEM) images of (AgNPs) (scale bar = 100 nm). **C** High-resolution TEM image of Ag nanoparticle (scale bar = 20 nm)

**Fig. 6:**
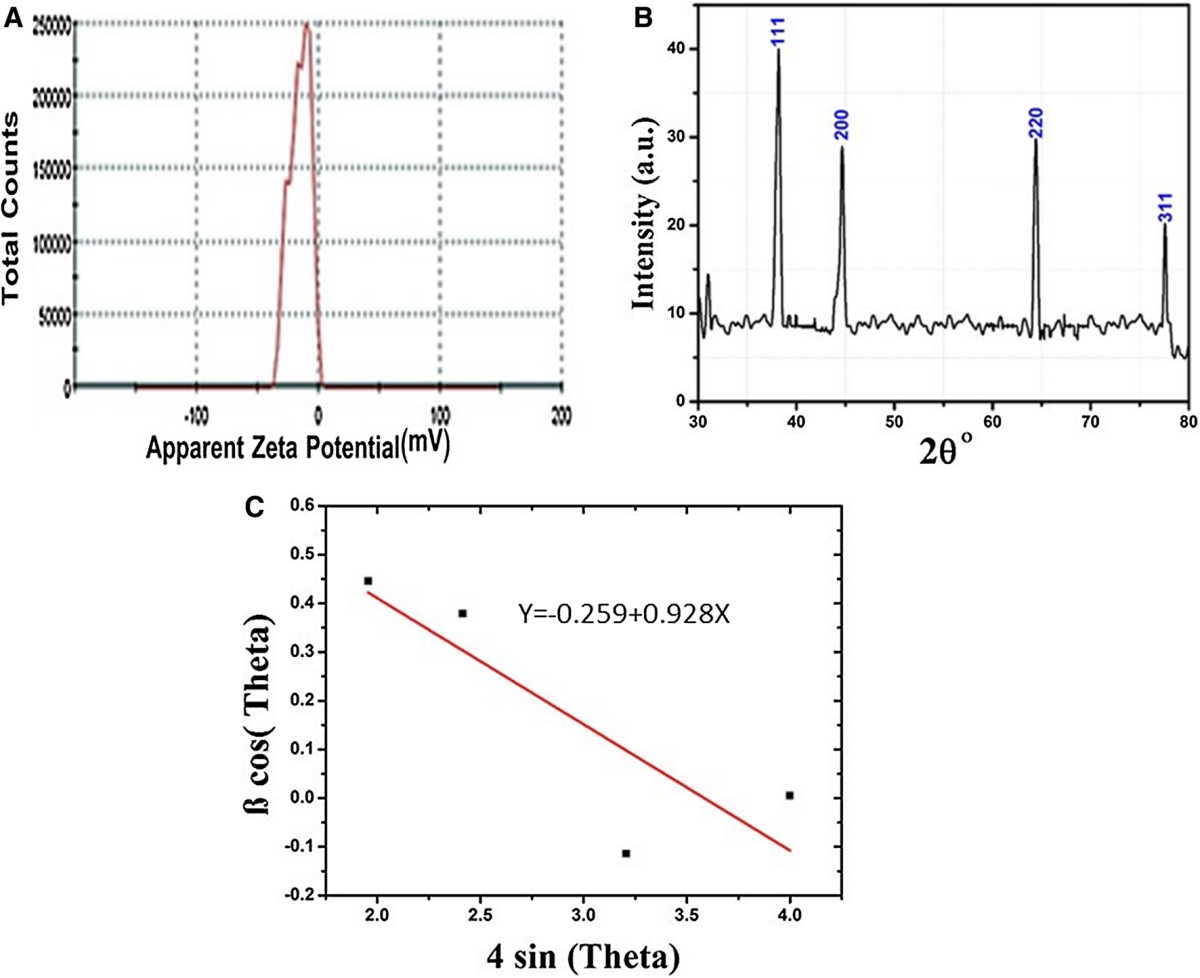
Physical characterization. **A** Zeta Potential of biosynthesized AgNPs by *Haloferax sp* strain NRS1. **B** X-ray diffraction pattern of biosynthesized AgNPs by *Haloferax* sp strain NRS1. **C** Williamson–Hall analysis of *Hfx*-Ag nanoparticles. The slope and crystalline size from the intercept of the fit were used to determine strain lattice

TEM investigation was conducted to determine *Hfx*-AgNPs size, as shown in Fig. 5. The shape of Ag nanoparticles synthesized by *Haloferax sp* strain NRS1 is mostly spherical; and the average particle size is 27.7 nm. The diameter range is 5.77- 73.14 nm. Geometric mean equal value of 24.96 ± 1.65 nm. The stability of *Hfx*-AgNPs was evaluated in terms of zeta potential (Fig. 6A). It was found that the zeta potential value was negative, i.e., − 25.5 ± 3.15 mV, which implies the good colloidal nature of synthesized nanoparticles.

Figure 6B shows the powder XRD pattern of the *Hfx*-AgNPs. The diffraction peaks appeared at four 2θ angles equal to 38.21°, 44.63°, 64.39° and 77.61° which correspond to (hkl) planes (111), (200), (220) and (311), respectively. The crystallite of *Hfx*-AgNPs sizes was 29.28 nm as calculated using Debye Scherrer's equation. The full width at half maximum (FWHM) values of the nanoparticles was consistent with their size and was ranged from 0.190 to 0.511 (Table 1). Lattice strain in the biosynthesized AgNPs by *Haloferax sp* strain is determined from the slope W–H plot (Fig. 6C), which equal to − 0.259 ± 0.114.

**Table 1:** The crystalline size of Silver Nano-powder synthesized by *Haloferax sp* strain NRS1

Peak position (2θ°)	Hkl	FWHM Radians (β)	Amplitude	Crystalline Size of the particles (D, nm)	β cos θ	4 sin θ
38.21	111	0.511	16.788	17.197	0.446	1.956
44.63	200	0.475	9.655	18.897	0.379	2.413
64.39	220	0.393	9.391	24.968	0.0050	3.999
77.61	311	0.190	− 2.097	56.082	− 0.114	3.206
			(D) Average	29.28		

**Fig. 7:**
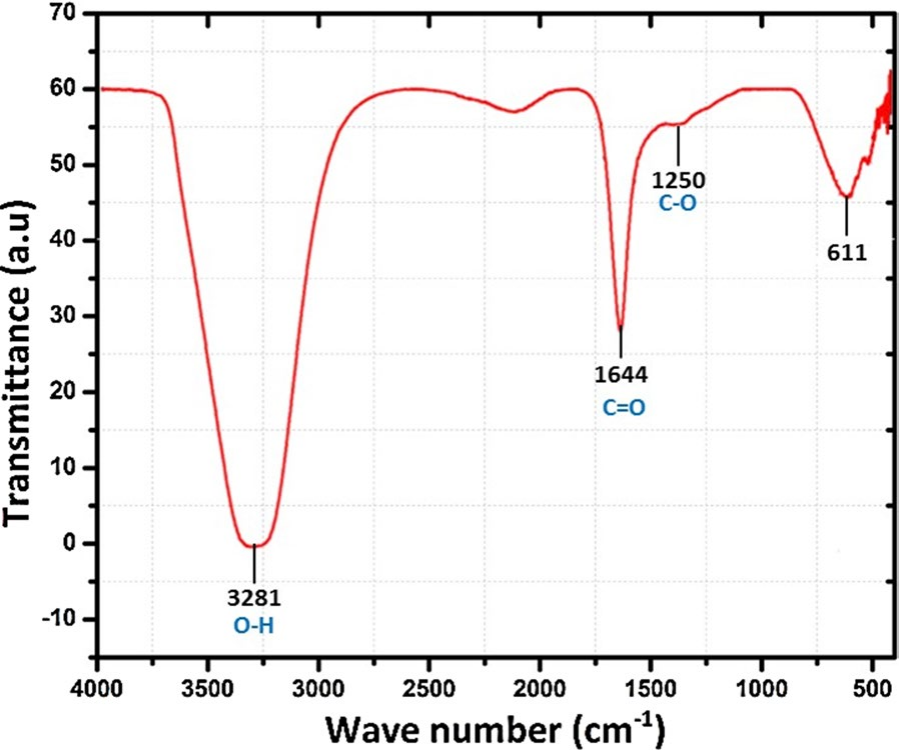
FTIR spectra of *Hfx*-AgNPs

The FTIR spectrum showed characteristic absorption frequencies of the O–H stretching group at 3281 cm^−1^ wavelengths, which probably present either in phenol or alcohol. The C=O stretch of carbonyl groups was observed at 1644 cm^−1^, which was related to amino acid residues and peptides and had a strong capability to bind to metal nanoparticles. Furthermore, C–O stretching vibrations were determined at 1250 cm^−1^, being related to aromatic esters, where Ag_2_O has a stretching vibration; Ag_2_O may form at Ag nanoparticles' surface (Fig. 7).

### Bioactivity of silver nanoparticles

**Fig. 8:**
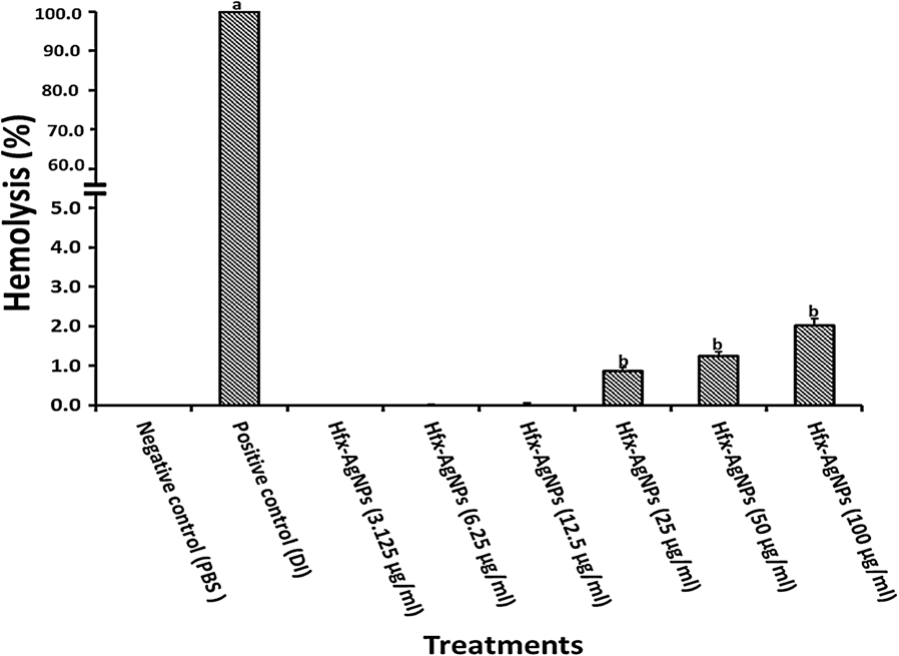
Hemolysis assay for *Hfx*-AgNPs colloidal solution against human erythrocytes, using DI as a positive control (+) and PBS as a negative control (−). Different concentration of *Hfx*-AgNPs were incubated with RBCs for 60 min for determining hemolytic activity. Data is represented as mean ± SD (n = 3). The mean difference between treatments considered significant at p < 0.05 using a one-way analysis of variance followed by Duncan's post hoc test. ^a^ statistically significant difference when compared with negative control; ^b^ statistically significant difference when compared with positive control

All materials that enter the blood get in contact with red blood cells (RBC). A hemolysis test was accomplished to assess *Hfx*-AgNPs impact on erythrocyte by measuring hemoglobin release after exposure to various concentrations of *Hfx*-AgNPs colloidal solution. The hemolysis assay performance was tested by the negative control PBS and positive control deionized distilled water (Fig. 8). It was observed that the hemolytic percentage of *Hfx*-AgNPs was concentration-dependent. Hemolysis was not detected at concentrations below 12.5 µg/ml, while 2.01% hemolysis was seen at the concentration of 100 µg/ml. According to the ISO/TR 7405-1984(f), the samples were considered as hemolytic if the hemolytic percentage was above 5%.

**Fig. 9:**
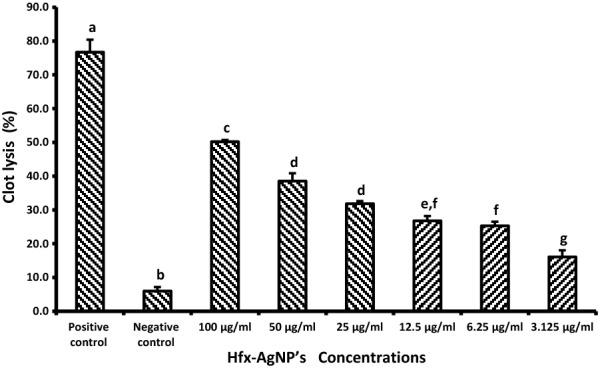
In vitro thrombolysis activity of *Hfx*-AgNPs. Positive control- streptokinase (SK), Negative control (saline solution). Data are expressed as mean ± SD (n = 3). The mean difference between different *Hfx*-AgNPs concentrations was considered significant at p < 0.05 using a one-way analysis of variance followed by Duncan's post hoc test for multiple comparisons. Lowercase letters compare the positive and negative control with different concentrations of *Hfx*-AgNPs colloidal solution. Same lowercase letters mean no statistically significant difference. However, Different lowercase letters mean a statistically significant difference

The effectiveness clot lysis by *Hfx*-AgNPs, positive thrombolytic control (streptokinase), and negative control is represented in Fig. 9. The addition of 200 µl SK, a positive control to the clots at 37 °C, showed 76.7% clot lysis. When treated with a saline solution (negative control), clots showed only negligible clot lysis (6.0%). The mean difference in clot lysis percentage between positive and negative control was significant (p value < 0.05). After treating clots with different concentrations of *Hfx*-AgNPs colloidal solution, the highest percentage of clot lysis was displayed at 100 μg/ml of *Hfx*-AgNPs with 50.218%, which means the percentage of the clot lysis compared with the negative control was significant (p-value < 0.05). Also, it has been observed that the lysis activity of tested agent was dose-dependent.

## Discussion

The results of the present study demonstrated the biosynthesis of silver nanoparticles mediated *Haloferax sp* strain NRS1. The brown-black color of the culture confirmed the reduction of silver ions (Ag^+^) with the formation of AgNPs, according to Abdollahnia et al. (2020). Silver nanoparticles synthesis by *Hfx. sp* strain NRS1 was potential occurred in the presence of 0.5 mM AgNO_3_, 25% NaCl (w/v), at 37 °C. The nanostructures' size, shape, and distribution are related to the intensity and frequency of the plasmon surface's absorption bands (Petryayeva and Krull, 2011). Sharp peaks in silver nanoparticles' spectral extinction at visible and near-infrared frequencies rely on the resonances of localized surface plasmon (Mayer and Hafner, 2011). In the wavelength range of 300–800 nm at a resolution of 1 nm (Peiris et al. 2017). Owing to the elevated optical density (OD) of the colloidal suspension, a 1 ml colloidal aliquot was diluted with 15 mL of distilled water. Distilled water was used as blank. The analysis of *Hfx*-AgNPs colloidal solution by UV–Vis spectroscopy showed a characteristic absorbance peak at 458 nm, mostly similar to those for AgNPs synthesized by *Halococcus salifodinae* with maximum absorption peaks at 440 nm (Srivastava et al. 2013). Also, a peak equal to 446 nm was demonstrated for colloidal nanoparticles synthesized by *Haloferax denitrificans* (Abdollahnia et al. 2020).

### Transmission electron microscopy (TEM)

The size and morphology of *Hfx*-AgNPs were done using TEM. Typically, the TEM pictures of 10 replicates of tested samples were taken at different 3 magnifications (30,000, 80,000, and 100,000 X). A drop of aqueous AgNPs sample was placed on a carbon-coated copper grid. The samples were allowed to dry under an infrared lamp before the examination; the micrographs were obtained using TEM operating at an accelerating voltage of 200 kV (Schrand et al. 2010). Dimension and morphology of *Hfx*-AgNPs were performed using the threshold method after set scale than the "Analyze Particle "feature to measure each particle area and diameter (Woehrle et al. 2006; Schneider et al. 2012). The particle size has been measured using image analysis. The present results confirmed the spherical-oblate shape of biosynthesized nanoparticles by *Haloferax* sp strain NRS1, in size ranges from 5.77 to 73.14 nm. The average particle size for AgNPs synthesized by *Halococcus salifodinae* was found to be around 50.3 nm (Srivastava et al. 2013).

### Zeta potential analysis

Zeta potential determination is an essential and easiest means of predicting the stability and understanding of nanoparticle surface quality (Gregory et al. 2016); which displays the magnitude of repulsions between adjacent, equally charged dispersed particles whose values are correlated to colloidal dispersion stability (Larsson et al. 2012). the aqueous suspension of *Hfx*-AgNPs was placed in a DTS0112-low volume disposable cuvette. The potential of zeta is determined by the electrophoretic mobility principle, induced by applying an electric field through dispersion media. The silver nanoparticles have carried the charge of capping agents (Singh et al. 2014). The current investigation results revealed that; zeta potential shows negative value (− 25.5 ± 3.15 mV) for the *Hfx*-AgNPs reaction mixture. The negative values of zeta potential analysis confirmed the stability of *Hfx*-AgNPs. As formulated by Meléndrez et al. (2010), the magnitude of zeta potential gives an implication of colloid's possible stability. Similar observation revealed that; zeta potential with a negative value equal to − 18.9 mV is considered stable (Salvioni et al. 2016). The zeta potentials of silver nanoparticles produced by other halophiles such as *Haloferax sp.* and *Halomonas* sp isolated from solar saltern were equal to − 33.12, − 35.9, respectively (Gregory et al. 2016). These data confirmed that the haloarchaeon *Haloferax* sp strain NRS1 used in the present study produced the nanoparticles which are comparably stable as previously synthesized silver nanoparticles.

### X-ray diffraction (XRD)

Dried *Hfx*-AgNPs were coated on an XRD grid to determine their structural characteristics using an XRD analysis. X-ray diffractometer was recorded by monochromatic Cu kα radiation (λ = 1.78 Å) with TD-2500 XRD which ran at 40 kV. The scanning was measured in the region of 30°–80°. For 2θ (Bragg diffraction angle) at 0.02°/min, and the time constant was 2 s (Klung and Alexander 1962). FWHM (full width at half maximum) of peaks was determined by Origin pro using the "Quick fit" command. Then the crystallite size was calculated using the Debey-Scherrer equation (Eq. 1) (Holzwarth and Gibson 2011):

1 $$ FWHM = \frac{K\lambda }{{L{\text{Cos}} \theta }} $$

L is particle size, θ is peak position (2θ/2) in radian. λ is the wavelength of the X-ray diffraction. K = 0.89 is the Debye–Scherrer constant. The lattice strain, ε, were estimated using the Williamson–Hall (WH) plot, determined according to Singaravelan and Alwar (2015) using the following equation (Eq. 2).

2 $$ \beta \cos \theta = \frac{C\lambda }{T} + 4\varepsilon \sin \theta $$

where β is FWHM in radian, *t* is the grain size in nm, ε is the strain, and C is a correction factor taken as 0.94.

The XRD peaks of *Hfx*-AgNPs exhibited a strong and narrow pattern, implying that nanomaterials synthesized by *Haloferax sp* strain NRS1 displayed a high degree of crystallinity. Peaks from other impurities were not detected, which confirming the high purity of the synthesized AgNPs. XRD provided significant evidence for the complete reduction of silver ions resulting in nanomaterials' production (Bhainsa and D'souza 2006). The XRD patterns of silver nanoparticles produced by archaeal strain *Haloferax sp* strain NRS1 showed an unassigned peak at 38.21°, 44.63°, 64.39°, and 77.61°. Thus, the XRD pattern clearly illustrated that the Ag-NPs formed in this study were crystalline in nature. According to the previous study, the peaks corresponding to our findings confirm that the main crystalline phase was silver (Sadhasivam et al. 2010; Shameli et al. 2012; Gurunathan et al. 2013). Concerning the FWHM data, the silver nanocrystalline structure obtained by haloarchaeal strain *Haloferax sp*. NRS1 (29.28 nm), which is similar to a previous report for NPs by the halophilic strain, *Halococcus salifodinae* BK 3 (22 nm) (Srivastava et al. 2013). In comparison with our previous work at Sayed et al. (2018) who investigate the characterization of nano silver powder (Sigma Aldrich, 576832) and nano silver ink (Metalon, JS-B25HV) X-ray diffraction pattern revealed four peaks 111, 200, 220, 311, these results were in accordance with our findings. Approving the formation of silver nanoparticle by the studied strain NRS1.

### Fourier Transmission Infra-Red (FT-IR) spectroscopic

For analysis of FTIR of *Hfx*-AgNPs, the colloidal solution was centrifuged at 10,000 rpm for 30 min. The pellet was washed three times using deionized water to eliminate the free proteins and enzymes. Then a small quantity of dried powder was grinded with potassium bromide (KBr). The sample's FTIR spectrum was recorded at 400–4000 cm^−1^ (Taran et al. 2016). The results of FTIR confirmed the capping agents of some functional groups responsible for the biosynthesized nanoparticles' stability (Ajitha et al. 2018). The bands were observed at 3281 cm^−1^, 1644 cm^−1^, 1250 cm^−1^ and 611 cm^−1^. The current result confirmed that the amide group from amino acid residues and peptides of proteins has a stronger ability to bind to metal. The amino group could form a coat covering the metal nanoparticles to stabilize silver nanoparticles formed intracellularly. This evidence suggests that the biological molecules could perform the function of stabilizing the intracellular biosynthesized nanoparticles (Soenen et al. 2010). According to Makarov et al. (2014), the silver nanoparticle surface proteins act as capping agent amino acid residues. Peptides have a strong ability to bind to silver ions. Comparing the present findings with our previous work, Sayed et al. (2018), FTIR of nano silver powder (Sigma Aldrich, 576832) and nano silver ink (Metalon, JS-B25HV) revealed presence of O–H stretch, C=C stretch and N–H groups which are almost similar to *Hfx-*AgNPs composition.

### Bioactivity of silver nanoparticles

Previous studies reported the potential antimicrobial activity of synthesized silver nanoparticles by *Haloarchaea* (Srivastava et al. 2014; Patil et al. 2014; Abdollahnia et al. 2020). The current study reports new bioactivities for green synthesized silver nanoparticles by *Haloferax* strain. Hemolytic activity against erythrocytes determines the tested material's potential membrane-stabilizing capability (Seeman and Weinstein 1966). The results of the present study revealed significant membrane-stabilizing potential associated with low toxicity of *Hfx*-AgNPs. This finding is supported by the negative value recorded for *Hfx*-AgNPs zeta potential. The nanoparticles' negative charge may prevent RBCs from interacting with the nanoparticles, consequently (Rothen-Rutishauser et al. 2006). Thus, *Hfx*-AgNPs may display their favorable hemolysis rate due to carboxylate group presence, leading to repulsion with RBC. Our results are in accordance with Singh et al. (2020). They stated that Fe_3_ O_4_ -Au Cys and Fe_3_ O_4_ -Au Cyt nanoparticles show low hemolytic activity due to their negative charges related to the presence of functional groups with a negative charge, which may lead to repulsion with RBC. According to Guidance for Industry and Food and Drug Administration Staff (FDA)-2013-D-0350 (ISO 10993-1) protocol, when the hemolysis level is above 5%, the tested material is considered a hemolytic agent.

At the present time there is an urgent need to discover drugs to break up or remove blood clots, as the formation of clots is the main cause of heart disease and stroke (Almalk and Zhang 2020). Regarding clot lysis assay, negative control revealed that clot dissolution did not occur when saline was added to the clot. However, significant thrombolytic activity was observed after treating the clots with *Hfx*-AgNPs colloidal solution as compared with the negative control. The *Hfx*-AgNPs thrombolysis activity was compared with streptokinase. The current result identified the potent thrombolytic activity of the tested material. According to Marder et al. (2001), thrombolytic agents dissolve blood clots by activating plasminogen that converted to a proteolytic enzyme called plasmin. Plasmin is capable of breaking cross-links between fibrin molecules, which provide the structural integrity of blood clots. Recently Colasuonno et al. (2018) summarize that some nanotherapeutic agent exerts its action through tissue plasminogen activation, maximizing thrombolytic activity. A variety of nanoparticles with clot-specific targeting capability release some thrombolytic agents (Landowski et al. 2020). Moreover Deng et al. (2018) stated that fibrin-targeted nanoparticle considered as promising drug delivery system which improve systemic hemostasis in vivo.

To sum up, the haloarchaeon *Haloferax sp* strain NRS1 (MT967913), isolated from solar saltern in Jeddah, Saudi Arabia, can accumulate silver in a nanoparticle form. AgNPs biosynthesized in NTYE medium with an average particle size equal to 27.7 nm. The biosynthesis of silver nanoparticles was confirmed using UV–visible spectroscopy and TEM, Moreover, FTIR showed that *Hfx*-AgNPs considered as both reducing and stabilizing agent. X-ray diffraction analysis revealed that the AgNPs were crystalline in nature. The relatively stable nature of *Hfx*-AgNPs could be attributed to their zeta-potential value, which displayed a realistic surface charge magnitude. *Hfx*-AgNPs exhibited low hemolytic activity, which may be related to negative zeta-potential, confirming their promising role as nano-drug delivery agents. However, increased clot lysis properties may be attributed to the activation of the clot lysis's cascade reaction.

## Data Availability

Available upon request.

## Abbreviations

AgNPs: Silver nanoparticles
Hfx-AgNPs: Silver nanoparticles biosynthesized by Haloferax sp
TEM: Transmission Electron Microscopy
XRD: X-ray Diffraction
FT-IR: Fourier Transmission Infra-Red
RBC: Red blood cells
FWHM: Full width at half maximum
